# COVID-19 in Pediatric Inpatients: A Multi-Center Observational Study of Factors Associated with Negative Short-Term Outcomes

**DOI:** 10.3390/children8110951

**Published:** 2021-10-22

**Authors:** Sara Rubenstein, Emily Grew, Katharine Clouser, Alexander Kwok, Aravindhan Veerapandiyan, Jeffrey Kornitzer, Keith Pecor, Xue Ming

**Affiliations:** 1 Department of Neurology, Rutgers New Jersey Medical School, Newark, NJ 07103, USA; Sara.Rubenstein@hmhn.org (S.R.); ecg81@njms.rutgers.edu (E.G.); jkornitzer@gmail.com (J.K.); 2 Pediatric Hospital Medicine, Hackensack University Medical Center, Hackensack, NJ 07601, USA; katharine.clouser@hmhn.org; 3 Department of Pediatrics, Hackensack Meridian School of Medicine, Nutley, NJ 07110, USA; 4 Division of Pediatric Neurology, University of Arkansas for Medical Sciences, Little Rock, AR 72005, USA; askwok@uams.edu (A.K.); aveerapandiyan@uams.edu (A.V.); 5 Division of Neurology, New Jersey Pediatric Neuroscience Institute, Morristown, NJ 07960, USA; 6 Division of Child Neurology, St. Joseph’s Children’s Hospital, Paterson, NJ 07503, USA; 7 Department of Biology, The College of New Jersey, Ewing, NJ 08628, USA; pecor@tcnj.edu

**Keywords:** COVID-19, age, body mass index, pediatric intensive care unit, respiratory support

## Abstract

Most cases of COVID-19 in children and adolescents are mild or asymptomatic, but a small number of individuals may develop severe disease, requiring PICU admission and/or mechanical ventilation. We assessed the factors associated with negative short-term outcomes of COVID-19 in 82 pediatric patients at three hospitals within the United States during the spring and summer of 2020 using medical records, laboratory data, and imaging studies of all patients admitted with a positive RT-PCR test for SARS-CoV-2. We found that older patients were more likely to have an extended hospital stay, and those with high BMIs (over 25) were more likely to be admitted to the PICU during the early pandemic. In addition, older patients, those with high BMIs, and those with underlying medical conditions, were more likely to receive respiratory support. Given the association of age, BMI, and underlying medical conditions with more severe COVID-19, clinicians should keep these factors in mind when treating patients.

## 1. Introduction

Pediatric COVID-19, the disease caused by the SARS-CoV-2 virus, has been ubiquitously reported in the United States [1]. During the first wave of the pandemic, it became apparent that children with COVID-19 are largely asymptomatic or have very mild symptoms, and that severe illness among children is rare [2,3]. A small percentage of children do develop severe symptoms, even requiring admission to the pediatric intensive care unit (PICU) or utilization of mechanical ventilation [4,5]. A European multi-center cohort study published in September 2020 found that 8% of pediatric COVID-19 patients required PICU admission, and 4% required mechanical ventilation [3]. Of those children, one-quarter had pre-existing medical conditions. A large multi-centered study of 874 hospitalized patients by Bhalala, et al. found that 46.3% were admitted to the PICU, of which 48.0% had comorbidities [5]. Sixteen of these patients died, equating to a hospital mortality rate of 1.8%. Data derived from the early pandemic suggest that Black and Hispanic children and children from low-income households have an increased risk of SARS-CoV-2 infection [6]. Further evidence suggests that some children, especially those with underlying medical conditions and other risk factors, may be at increased risk of severe disease or even death. In this study, we present an assessment of possible factors related to negative short-term outcomes for pediatric patients admitted to three hospitals in the United States during the first wave of the COVID-19 pandemic, and references in this study were primarily drawn from the studies performed during similar periods of the pandemic.

## 2. Materials and Methods

### 2.1. Study Design and Data Collection

This retrospective, multi-center, and observational study was conducted simultaneously at—and in collaboration with—St. Joseph’s Children’s Hospital in Paterson, New Jersey; Joseph M. Sanzari Children’s Hospital at Hackensack University Medical Center in Hackensack, New Jersey; and Arkansas Children’s Hospital in Little Rock, Arkansas. All pediatric patients aged up to 21 years from 15 March through to 30 July 2020 were reviewed, resulting in a study duration of 21 weeks. All patients with positive results for SARS-CoV-2 reverse transcriptase polymerase chain reaction (RT-PCR) assay of nasopharyngeal swabs were considered for analysis. At the time of our study, serum antibody assays were deemed unreliable and were not performed on most subjects.

The electronic medical records, laboratory data, and imaging studies for all patients admitted to the hospitals with laboratory-confirmed SARS-CoV-2 infection were queried. Data on age, sex, ethnicity, BMI, and relevant medical history, including a documented history of, or treatments for, cancer, diabetes, sickle cell disease, epilepsy or known seizures, developmental delay, and other neurological conditions, as well as asthma and other chronic lung disease were collected. For each patient, presenting symptoms including respiratory, systemic, and neurological symptoms were recorded. Inflammatory markers, including white blood cell count, total lymphocyte count, liver function tests, and C-reactive protein (CRP) were analyzed. We reviewed all available lung imaging. Poor outcome secondary to SARS-CoV-2 infection was evaluated based on the length of hospital stay, PICU admission, and respiratory support required during hospital admission. Discharge from hospital or death was documented for each patient. Two children from Hackensack University Medical Center in this study were also included in the cited study by Feldstein et al. [7]. Four additional patients were included in a study by Bhavsar et al. [8].

This study was approved by the institutional review boards at each of the institutions. Informed consent was waived due to the retrospective nature of the study design.

### 2.2. Statistical Analyses 

The variable formats of our outcomes of interest (length of stay, PICU admission, and respiratory support) and our independent variables (age, sex, BMI, and relevant medical history) were not uniformly recorded for all patients among the three hospitals. As such, using a multiple regression model would have reduced our sample size dramatically, and we therefore chose to run individual logistic regressions with Wald statistics and odds ratios for each combination of independent variable and outcome. Statistical calculations were made using SPSS v.26.

## 3. Results

### 3.1. Demographics

Our dataset included the electronic medical records of a total of 83 pediatric patients aged zero to twenty-one years old admitted with both symptoms consistent with, and RT-PCR confirmed, COVID-19 between 19 March and 29 July of 2020. One patient from Arkansas Children’s Hospital was excluded from analysis as their severity of illness and admission to the PICU were attributable to epidural hematoma sustained in an ATV accident, with RT-PCR-confirmed COVID-19 not thought to have contributed to PICU admission. The remaining 82 patients presented with respiratory, neurological, or other symptoms suggestive of COVID-19, including abdominal pain and gastrointestinal distress. Most common physical exam findings included 34.8% of patients with respiratory distress, 19.6% with conjunctivitis, and 10.9% with altered mental status. These patients were admitted to the hospital with RT-PCR-confirmed COVID-19 and were included in the analysis. Their demographic information is summarized in Table 1. In addition to RT-PCR testing, 35 patients underwent SARS-CoV-2 antibody testing. Of those patients, 14 (40%) were positive for IgG antibodies; none were positive for IgM.

Our study had 10 patients with suspected MIS-C, with a median age of 4. The median BMI was 19.6, and an equal number were male and female. While the characteristics of MIS-C were not well established at the time of this study, the symptoms of these 10 patients were reviewed against later reported MIS-C clinical presentation and deemed to be consistent with MIS-C.

### 3.2. Length of Stay 

**Figure 1:**
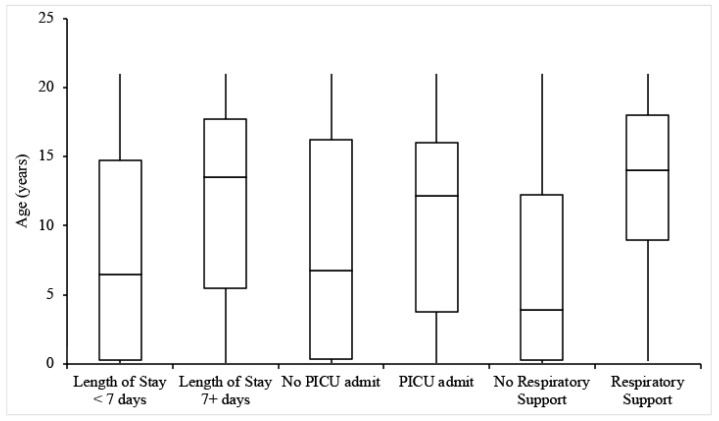
Box-and-whisker plots for the ages of patients experiencing each outcome of interest.

**Figure 2:**
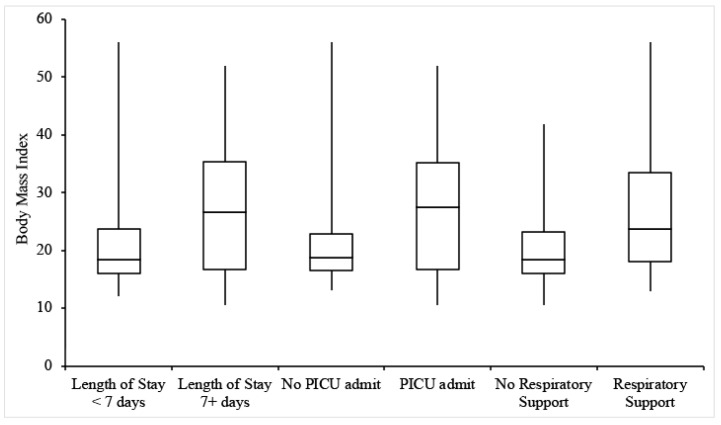
Box-and-whisker plots for the BMIs of patients experiencing each outcome of interest.

Twenty-two patients (26.8%) were admitted for at least 7 days. The incidence of a length of stay of 7+ days increased with patient age but was not dependent on sex, BMI, or medical history (Table 2 and Table 3, Figure 1 and Figure 2). MIS-C patients had an average length of stay of 4.2 days, and MIS-C patients admitted to the ICU had an average stay of 4 days, both less than the overall group. Only 1 MIS-C patient stayed for longer than 7 days.

### 3.3. PICU Admission 

Thirty-four patients (41.5%) were admitted to the PICU, with the remainder being admitted to the General Pediatrics ward. Of note, the rate of 41.5% for PICU admission was derived from hospitalized patients. Individuals with higher BMIs were more likely to be admitted to the PICU, but there was no significant relationship between PICU admission and age, sex, or relevant medical history (Table 2 and Table 3, Figure 1 and Figure 2).

Of the 34 hospitalized patients admitted to the PICU, 12 were for hypoxia, 4 for shock or sepsis, and 4 for concern for MIS-C. Other reasons for admission were highly variable and included cardiac symptoms such as bradycardia or atrial fibrillation, neurologic symptoms, and gastrointestinal symptoms.

Some patients in the PICU experienced severe outcomes including venous thrombosis, requirement of vasoactive substances, and death. Among PICU admits, 31.3% required vasoactive substances including dopamine, norepinephrine, and milrinone. In our sample, five patients (15.2% of PICU admits) developed venous thrombosis; two patients with pulmonary emboli, two with central nervous system/venous sinus thrombosis, and one experienced deep vein thrombosis. The median age of these patients was 16 and the median BMI was 31.0. Four of the five patients were Hispanic or Latino; three were male. Three patients had no pre-existing medical history. One patient had a history of cancer and presented with jaundice and scleral icterus; this patient presented with a white blood cell count of 194.2 × 10^3^/microliter, and it was unclear whether the cancer was active, treated, or in remission. This patient was the sole death in our study, resulting in a mortality rate of 1.2%. Another patient with a history of syncope during menstruation had atrial fibrillation on presentation. Two patients who developed venous thrombosis required ventilator support, one being the patient with a history of cancer, and the other with no medical history. No patients in the sample were reported to have required ECMO or cardiac resynchronization therapy (CRT).

Among MIS-C patients, PICU admission rate was 40%. While the PICU admission rate was similar to the overall group, no MIS-C patients experienced adverse events including venous thrombosis, acute respiratory distress syndrome (ARDS), secondary bacterial pneumonia, or death. Five out of ten MIS-C patients required vasopressor support.

### 3.4. Respiratory Support and Imaging

Twenty-nine patients (35.5%) received respiratory support during their hospitalization, 18 of whom were admitted to the PICU. Types of respiratory support included nasal oxygen, non-rebreather mask, opti-flow/high flow oxygen, and mechanical ventilation. There was a positive relationship between receipt of respiratory support and both age and BMI (Table 2 and Table 3, Figure 1 and Figure 2). In addition, individuals with relevant pre-existing medical history were more likely than those without to receive respiratory support (Table 2 and Table 3). Common medical conditions among these patients included epilepsy (9.9% of total patients and 25% of those with relevant medical history), developmental delay (7.3%/18.2%), cancer (4.9%/12.5%), and immunosuppression (8.3%/17.5%). Of the 10 patients with a history of neuro-developmental disorders (including developmental delay, epilepsy, cerebral palsy, and neuromuscular disorders), 70% received some level of respiratory support. Four of the five patients requiring mechanical ventilation had underlying comorbidities. Receipt of respiratory support was independent of sex. One patient under the age of 5 years, with no past medical disorders, was excluded from this analysis due to missing information. Of MIS-C patients, 2 out of 10 required respiratory support; one patient received nasal oxygen, and another received opti-flow/high flow oxygen. Patients receiving respiratory support developed venous thrombus at a rate of 15.2%, compared to 6.1% of all patients experiencing this outcome.

Eighteen patients (30.0%) had abnormal results on chest imaging. Of these, 14 (77.8%) had pulmonary infiltrates, 8 (44.4%) had ground glass opacities, and 2 (11.1%) had pleural effusion. Twenty patients (24.4%) had normal imaging results, while in 44 patients (54.0%), chest imaging was not indicated and therefore not performed. Patients with abnormal chest imaging results were older, with a median age of 16, and had a median BMI of 29.3. Patients with abnormal chest imaging developed ARDS at a rate of 16.67% and secondary bacterial pneumonia at a rate of 27.5%, compared to 3.7% and 17.4% of all patients, respectively. Three out of ten patients with MIS-C had abnormal chest imaging results; two had pulmonary infiltrate alone, while one had pulmonary infiltrate and ground glass opacities.

## 4. Discussion

We found that higher BMIs, older age, and relevant medical conditions were associated with more severe COVID-19 in this cohort of hospitalized children during the first wave of COVID -19 in the United States. Higher BMI was associated with PICU admission and need for respiratory support, and older age was associated with longer length of stay in the hospital, as well as need for respiratory support. We also found that children with relevant medical conditions were at increased risk of requiring respiratory support. Conversely, younger children, those with lower BMIs, and those without comorbidities had less severe COVID-19.

### 4.1. Age

In our study population, older age was associated with longer hospital stay and the need for respiratory support. Patients admitted to the PICU also had a median age nearly twice that of those admitted to the General Pediatrics ward, although this relationship was not statistically significant. Similarly, a study at the Children’s Hospital at Montefiore found that 84% of patients admitted to the PICU were age 11 or older [9]. At New York-Presbyterian Morgan Stanley Children’s Hospital, the median age of all admitted patients was nine, while that of children admitted to the PICU was 14 [10]. Bhalala, et al. similarly found an older median age for their PICU patients (10.0) compared to their non-PICU patients (5.67) [5]. In contrast, among 582 children testing positive for COVID-19 across 25 European countries, Gotzinger et al. reported that patients who required PICU admission were younger than those who did not [3]. Children less than 1 month old were significantly more likely to be admitted to the PICU. However, in their study, all of four patients with a fatal outcome were older than 10 years old. Finally, Graff et al. found that children at age extremes (<3 months or >20 years) were at increased risk of hospital admission and requiring respiratory support [11]. Our study found that the most represented groups among admitted patients were the youngest (<1 year old) and oldest (15–21 years old); only older age was statistically significant for increased risk of more severe COVID-19 infection.

Among individuals in our study, younger children were less likely to be admitted to the PICU, require respiratory support, or remain in the hospital for more than one week. It is known that older adults are at higher risk for severe COVID-19 disease compared to younger adults and children. However, it is unclear why younger children may be at lower risk compared to older children and adolescents. Dhochak et al. suggested that children may have more protective defenses against infection through trained immunity, by which innate immune cells are functionally re-programmed to a more active state following initial antigen stimulation [12]. This initial stimulation in children may be due to vaccinations, as suggested by the BCG vaccine’s protective effect against severe COVID-19 disease in both the elderly and children, or infection with other coronaviruses, which are a common cause of URI in pediatric populations [12]. Additionally, they noted that younger patients have a stronger lung regenerative capacity than adults, and that thrombotic events may be less common in the pediatric populations, placing children at lower risk of morbidity and mortality from SARS-CoV-2 infection [12]. Though these hypotheses were initially proposed to explain why children have less severe infection compared to adults, they may be applicable to younger children as compared with older children and adolescents as well. Overall, there is variability in the available pediatric literature concerning the association between age and COVID-19 disease severity, warranting further investigation. It is also worthwhile to consider that older children may do worse given that they may have higher BMIs and more comorbidities compared to younger children. We found a positive relationship between age and BMI (Pearson correlation, *r* = 0.64, *p* < 0.001) for the individuals in our sample. Additionally, children with a notable past medical history had higher median ages than those without past medical history, suggesting that these variables may modify the relationship between age and poor short-term outcome.

In our sample, five patients developed venous thrombosis. These patients were older and had higher BMIs than the overall group, most were Hispanic or Latino, and more than half had no past medical history. The outcomes of these patients suggest that those who are older and have elevated BMIs may be at risk of the serious outcome of venous thrombosis, regardless of medical history.

Further, we want to bring attention to the commonly discussed outcome of SARS-CoV-2 infection in children of multisystem inflammatory syndrome in children (MIS-C) [7,13,14]. Our study found 10 patients (12.2%) admitted with concern for MIS-C. Contrary to the reviews by Abrams et al. and Kornitzer et al. [13,14], children developing MIS-C were younger among our patient population and had relatively mild courses of disease, including shorter lengths of stay and fewer adverse outcomes. They were admitted to the PICU at a rate similar to that of the entire patient group. The median age of 4 for our MIS-C patients was also younger than that described by Feldstein et al. (8.2), and the rate of PICU admission for MIS-C patients in our cohort (40%) was less than that of their study (80%) [7].

### 4.2. BMI

Obesity and elevated BMI have been extensively reported as a predictor of severe infection in adult patients with COVID-19. One study from Shenzhen, China, conducted early in the pandemic, found that patients with obesity had a 142% increased risk of developing severe pneumonia [15]. In a larger study from New York City, published around the time of our data collection, high BMI was the second strongest independent predictor of hospitalization, after old age [15]. Large meta-analyses have demonstrated that obese patients had an increased prevalence of SARS-CoV2 infection, as well as more severe infection with increased rates of hospitalization, need for PICU admission, need for mechanical ventilation, and death [16,17]. A recent, large-scale study from Tripathi, et al. found that children with obesity were more likely to develop MIS-C and be admitted to the PICU (57%) [18]. Additionally, Bhalala et al. found that the median BMI of PICU patients was 20.1, compared to 18.9 in non-PICU patients [5].

Our findings were similar to those reported by the Children’s Hospital at Montefiore, where obesity was present in 30.4% of admitted patients [9]. However, only three patients (23%) of those admitted to their PICU were obese, while 38.2% of PICU patients in our study were either overweight (BMI between 25 and 29) or obese (BMI > 30). Similarly, a study at New York-Presbyterian Morgan Stanley Children’s Hospital found that 22% of their patients were obese, with an additional 16% being overweight [10]. Sixty-seven percent of children requiring mechanical ventilation had obesity as a comorbidity, compared to 20% in those not ventilated [10]. In our cohort, 60% of children requiring mechanical ventilation had elevated BMIs. Our results are also consistent with the Tripathi, et al. study in that children with obesity were more likely to be admitted to the PICU and experience severe illness [18]. However, only one (5%) of our patients with a BMI greater than 25 experienced MIS-C, compared to 35.7% of patients with obesity in their cohort. Our study agrees with Bhalala, et al. in that our PICU patients were older, however, the median age of our PICU-admitted patients was 27.6, compared to theirs of 20.1 [5].

Multiple hypotheses have been proposed as to why obesity contributes to more severe COVID-19 in adult patients, although there is less literature exploring this in the pediatric population. The same mechanisms underlying more severe infection in obese adult patients, such as chronic subclinical inflammation, impaired immune responses, and underlying cardiorespiratory diseases, can also be observed in children [19]. Respiratory physiology is impaired in obese adults, as well as children. Adipose tissue restricts movement of the respiratory muscles and causes decreased oxygen saturation, which may already be compromised in COVID-19 disease [20]. It has also been observed that obese children and adolescents have higher blood pressure, which may cause endothelial dysfunction [21]. As endothelial damage is thought to be one of the underlying mechanisms of the SARS-CoV-2 virus, this may place obese children at an increased risk of severe infection [19]. Obese patients, including children, have a chronic state of subclinical inflammation as well as changes to their immune systems including the numbers of cytokines and immune cells [22]. This pro-inflammatory state may place children at a greater risk of cytokine storming and more severe COVID-19 disease. Despite these hypothetical pathophysiologic constructs, the actual modulating effects of obesity on COVID-19 in the pediatric population remain to be elucidated.

### 4.3. Relevant Medical History

In our study, children with underlying medical conditions had an increased need for respiratory support. Association between PICU admission or length of stay and medical history was not statistically significant. We did not differentiate between underlying comorbidities in our analyses, and all patients with any medical history were included in this subgroup. The literature has shown that both adults and children with underlying medical conditions are at an increased risk of more severe COVID-19 disease [3,5,11,23]. In children, commonly reported comorbid conditions include asthma and respiratory diseases, diabetes, cancer, and epilepsy and other neurologic diseases [3,5,9,10,11]. In our study, only two patients had asthma, one had diabetes, and nine patients had a history of epilepsy. Our study, and those in the available literature, support that of children diagnosed with COVID-19, those with past medical history become sicker and require more care.

### 4.4. Ethnicity

Our study was not designed to test for differences in outcomes among ethnic groups, but we observed some possible trends that deserve attention in future work. First, length of stay was similar across ethnic groups. Whites/Caucasians had an average length of stay of 5.67 days, Hispanics/Latinos of 4.77 days, and African Americans of 5.89 days. Second, White/Caucasian and African American children were admitted to the PICU at lower rates, 25% and 36.4% respectively, than Hispanic/Latino children (47.2%). Finally, while 6.1% of all patients were ventilated, 18.2% of the 11 African American patients required ventilation. White/Caucasian children were ventilated at a rate similar to the overall group (6.25%), while the rate for Hispanic/Latino children was lower (2.7%).

Our study had more than twice as many Hispanic and Latino patients compared to any other racial group. Community health needs assessments performed by each hospital in 2019 suggest that Hispanic and Latino patients (45.1%) were overrepresented in our study compared to the hospitals’ populations served [24,25,26]. Black patients (13.4%) in our study were roughly consistent with the makeup of the populations served by the hospitals [24,25,26]. Most of the remaining patients were white (19.5%), at a proportion far lower than any of the hospitals’ reported white population served (42.8–63%). The remaining racial groups, Asian and Other, included three and four patients, respectively. An additional 11 patients did not have their racial background documented.

Goyal et al. found that after adjusting for age, sex, and socioeconomic status, Black and Hispanic children had an increased risk of COVID-19 positivity and known exposure [6]. Additionally, children of lower socioeconomic status were at a higher risk of infection. The Children’s Hospital at Montefiore and New York-Presbyterian Morgan Stanley Children’s Hospital both reported that Hispanic and Latino patients made up at least half of their admitted patients [9,10]. However, both studies identified Hispanic and Latino patients as constituting the majority of their hospital’s patient population, contrary to the reported populations served by the three hospitals in our study. In contrast, Bhalala et al. found that Hispanic patients, who made up 36.9% of their cohort, were admitted to the PICU (33.2%) at a rate significantly lower than non-Hispanic children (66.8%) [5].

### 4.5. Limitations

Limitations of our study include small sample sizes overall and incomplete data for some patients. Our patients represent a heterogeneous group including patients with MIS-C and without. There were a small number of MIS-C patients, thus our evidence is not strong enough to draw definitive conclusions about MIS-C. Given that the study was conducted in three separate institutions and no standard guideline was available in caring for COVID-19 at the time, documentation, data collection, and clinical management strategy may not have been uniform. Some children may have been admitted to the PICU at the beginning of the pandemic out of an abundance of caution. We were also limited by the nature of our work as a retrospective, observational study. Finally, age and BMI were correlated, and all else being equal, older individuals have more time to experience relevant medical history. This may have influenced some of our results.

## 5. Conclusions

We found that some pediatric patients did develop severe disease from SARS-CoV-2 infection early in the pandemic, and many of those hospitalized required PICU care or respiratory support, including mechanical ventilation. Older children, those with higher BMIs, and those with relevant medical histories were at an increased risk of more severe COVID-19. These factors should be considered when treating children and adolescents presenting with COVID-19, and should be compared to current trends to investigate the progression of COVID-19 in children from the early stages of the pandemic to the currently predominant Delta variant.

## Figures and Tables

**Table 1:** Sex, Age, Ethnicity, and Admitting Hospital for Subjects (N = 82).

Sex		Age (Years)		Ethnicity		Admitting Hospital	
Female	40 (48.7%)	<1	22 (26.8%)	African American	11 (13.4%)	Arkansas	22 (23.9%)
Male	42 (51.2%)	1–5	11 (13.4%)	Hispanic/Latino	37 (45.1%)	Hackensack	46 (56.1%)
		5–10	9 (10.9%)	White/Caucasian	16 (19.5%)	St. Joseph’s	14 (17.1%)
		10–15	16 (19.5%)	Asian	3 (3.7%)		
		15–21	24 (29.3%)	Other/Unknown	15 (18.3%)		

**Table 2:** Results from the logistic regressions.

Length of Stay	*n*	Wald	*p*	OR	OR 95% CI
Age	77	5.08	0.02	1.09	1.01–1.17
Sex	77	0.05	0.83	0.9	0.33–2.41
BMI	60	3.31	0.07	1.05	1–1.11
Medical History	77	0.35	0.56	1.35	0.5–3.67
**PICU Admission**	***n***	**Wald**	***p***	**OR**	**OR 95% CI**
Age	81	1.96	0.16	1.04	0.98–1.11
Sex	81	0.1	0.75	0.87	0.36–2.1
BMI	63	4.01	0.05	1.06	1–1.11
Medical History	81	0.51	0.48	1.39	0.56–3.42
**Respiratory Support**	***n***	**Wald**	***p***	**OR**	**OR 95% CI**
Age	81	11.41	0.001	1.13	1.05–1.21
Sex	81	0.6	0.44	0.7	0.28 1.74
BMI	63	4.86	0.03	1.07	1.01–1.13
Medical History	81	5.78	0.02	3.187	1.24–8.2

Abbreviations: PICU, pediatric intensive care unit; BMI, body mass index; OR, odds ratio; CI, confidence interval.

**Table 3:** Descriptive statistics for each outcome of interest.

	Length of Stay		PICU Admission		Respiratory Support	
	<7 Days	7+ Days	No	Yes	No	Yes
Age (years) ^a^	6.5	13.5	6.8	12.2	3.9	14.0
Sex						
Female	70.3%	29.7%	57.5%	42.5%	60.0%	40.0%
Male	72.5%	27.5%	61.0%	39.0%	68.3%	31.7%
BMI ^a^	18.5	26.7	18.8	27.6	18.5	23.7
Medical History						
No	73.9%	26.1%	62.5%	37.5%	75.0%	25.0%
Yes	67.7%	32.3%	54.5%	45.5%	48.5%	51.5%

Abbreviations: PICU, pediatric intensive care unit; BMI, body mass index. ^a^ Values reported are medians.

## Data Availability

Data is available upon request to corresponding author.
